# Weird animal genomes, sex and the evolution of new sex genes

**DOI:** 10.1186/1687-9856-2015-S1-O3

**Published:** 2015-04-28

**Authors:** Jennifer A Marshall Graves

**Affiliations:** 1 La Trobe University, Melbourne, VIC, Australia

## 

In humans and other mammals with XX females and XY males, the Y bears a gene (SRY) that induces testis differentiation in the embryo and switches on hormones that masculinize it. The human X has more than 1500 genes, but the tiny Y is a genetic wasteland and bears only 45 protein-coding genes, most active only in testis. To discover how human sex chromosomes got to be so weird, we compared the chromosomes, genes and DNA in distantly related mammals and even birds and reptiles (with completely different sex determining systems). Kangaroo sex chromosomes reveal the original mammal sex chromosomes, while the bizarre platypus sex chromosomes (more related to those of birds) tell us that human sex chromosomes and the SRY gene are relatively young. The human X and Y evolved from an ordinary chromosome pair as the Y degraded progressively. If Y degradation continues at this rate, it will disappear in just 5 million years. If humans don’t become extinct, new sex determining genes and chromosomes must evolve, maybe leading to the evolution of new hominid species.

Where will our new sex genes and chromosomes come from? Whereas mammals (and birds) have rather rigid systems, other vertebrates (particularly reptiles) show great variation in sex determining systems, and we can find many examples of switches in sex determining systems. Using a model of dosage-dependent and temperature dependent sex determination, we can readily understand switches between temperature-dependent and chromosome-dependent sex determination, and even between XY and WZ systems. We also see many ways in which genes or gene copies (often of the same genes which seem to be particularly good at this role) have taken on a sex determining function, and defined new sex chromosome systems.

